# Case Report: Unexpected long-term survival in metastatic anaplastic thyroid carcinoma through multidisciplinary management

**DOI:** 10.3389/fonc.2026.1943145

**Published:** 2026-09-14

**Authors:** Joonseon Park, Dong-Jun Lim, Jaekyung Lee, Sue Youn Kim, Chan Kwon Jung, Solji An, Kwangsoon Kim, Ja Seong Bae

**Affiliations:** 1 Division of Thyroid and Endocrine Surgery, Department of Surgery, College of Medicine, The Catholic University of Korea, Seoul, Republic of Korea; 2 Division of Endocrinology and Metabolism, Department of Internal Medicine, College of Medicine, The Catholic University of Korea, Seoul, Republic of Korea; 3 Department of Hospital Pathology, College of Medicine, The Catholic University of Korea, Seoul, Republic of Korea

**Keywords:** anaplastic thyroid carcinoma, BRAF wild-type, lenvatinib, long-term survival, multidisciplinary management, PD-L1, pembrolizumab

## Abstract

Anaplastic thyroid carcinoma (ATC) is one of the most lethal human malignancies, with a median overall survival of 3 to 5 months and a 1-year survival rate below 20%; survival beyond 2 years remains distinctly uncommon, particularly in patients with metastatic disease. We report a 45-year-old man who underwent left thyroid lobectomy for a mass initially attributed to an inflammatory condition; final pathology revealed an 8.0-cm ATC (pT3bN1a, Stage IVC). Molecular profiling demonstrated BRAF V600E and TERT promoter mutation-negative status, no actionable next-generation sequencing (NGS) targets, and an exceptionally high PD-L1 combined positive score (CPS) of 90. The postoperative course was complicated by progressive wound deterioration precluding radiotherapy. Following multidisciplinary team (MDT) consensus, lenvatinib (20 mg/day) plus pembrolizumab (200 mg every 3 weeks) was initiated on July 12, 2024; wound complications were managed through iterative surgical wound care conducted concurrently with uninterrupted systemic therapy over nine cycles. At the most recent follow-up on July 13, 2026 — 28 months after initial presentation — imaging demonstrated sustained disease control with no evidence of disease progression or newly developed lesions, and the patient remained alive under active surveillance, though with mild residual discomfort from skin adhesion at the prior drain site. This case demonstrates that durable disease control is achievable in selected patients with metastatic BRAF wild-type ATC, and highlights extreme PD-L1 expression as a potential biomarker guiding immunotherapy selection, alongside the critical importance of multidisciplinary coordination enabling treatment continuity despite significant surgical complications.

## 1 Introduction

Anaplastic thyroid carcinoma (ATC) is one of the most lethal solid tumors in clinical oncology. Despite representing only 1–2% of all thyroid malignancies, ATC is responsible for a disproportionate share of thyroid cancer–related mortality, with a historically reported median overall survival (OS) of 3 to 5 months and a 1-year survival rate below 20% (1, 2). Under the American Joint Committee on Cancer (AJCC) 8th edition staging system, all ATC patients are classified as Stage IV at diagnosis, reflecting the invariably aggressive and locally advanced or metastatic nature of the disease (3). Clinically, ATC typically presents with explosive growth over days to weeks, often with airway compromise, dysphagia, and vocal cord paralysis, rendering curative-intent surgery feasible in only a minority of patients.

The therapeutic landscape for ATC has evolved substantially since 2018. For the approximately 25–30% of patients harboring a BRAF V600E mutation, the combination of dabrafenib and trametinib demonstrated a confirmed overall response rate of 56%, leading to regulatory approval and establishing molecular-guided therapy as a paradigm in ATC management (4, 5). However, for the remaining majority with BRAF wild-type tumors, effective systemic options remain limited. Immune checkpoint inhibition has emerged as a promising strategy in this subgroup, supported by the observation that ATC tumors frequently exhibit high PD-L1 expression and a relatively immunogenic microenvironment (6, 7). Nevertheless, long-term survival in BRAF wild-type metastatic ATC remains exceedingly rare, and predictive biomarkers for durable response to immunotherapy-based regimens are not yet established. The 2021 American Thyroid Association (ATA) guidelines recommend comprehensive molecular profiling and rapid multidisciplinary team (MDT) engagement for all ATC patients at the time of diagnosis (1, 8).

Here we report a 45-year-old man with incidentally diagnosed metastatic BRAF wild-type ATC who achieved durable disease control beyond 2 years following multimodal treatment comprising left thyroid lobectomy, lenvatinib plus pembrolizumab, and iterative multidisciplinary surgical wound management. At the time of this report, he remains alive with an excellent performance status (ECOG 0). This case highlights the potential of extreme PD-L1 expression as a clinically actionable biomarker and the essential role of MDT-coordinated care in enabling treatment continuity in ATC. This report was prepared in accordance with the CARE reporting guideline, and the complete clinical course is summarized in the timeline (**Figure 1**).

**Figure 1:**
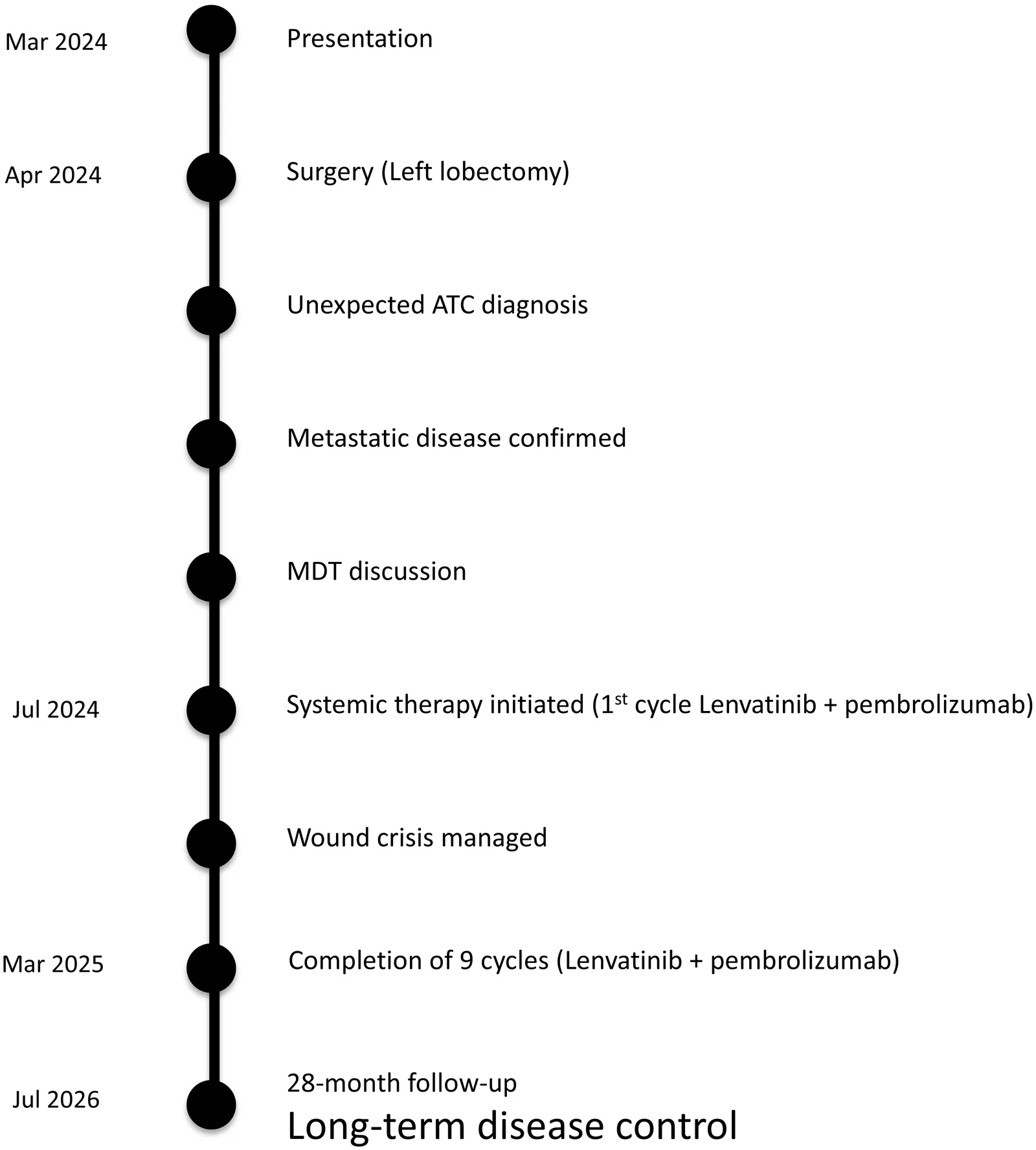
Clinical timeline. Timeline of key diagnostic, therapeutic, and follow-up events from initial presentation in March 2024 through the 28-month follow-up in July 2026, reflecting long-term disease control. ATC, anaplastic thyroid carcinoma; MDT, multidisciplinary team.

## 2 Case description

### 2.1 Initial presentation and diagnostic workup

A 45-year-old man with no significant past medical or family history was referred for evaluation of an enlarging left thyroid mass. The mass had been identified at an outside institution, and a previous needle biopsy had demonstrated chronic lymphocytic thyroiditis with fibrosis (Bethesda category II), interpreted as consistent with a benign inflammatory process. Contrast-enhanced neck CT (March 4, 2024) revealed a large (~4.7 cm), ill-defined, cystic and necrotic left thyroid mass with extrathyroidal extension to the ipsilateral strap muscles and sternocleidomastoid, perilesional fat stranding, and marked rightward laryngotracheal deviation. Given the unusually large size and atypical imaging features, the surgical team convened a multidisciplinary discussion, and additional cross-sectional imaging was recommended to further characterize the lesion. Neck ultrasonography (March 12, 2024) demonstrated a 39 × 39 × 43-mm heterogeneous left thyroid mass with focal calcification and possible abscess formation, and contrast-enhanced MRI (March 17, 2024) showed T2-heterogeneous signal intensity with peripheral multinodular soft tissue enhancement and marked diffusion restriction. On integrated review of all imaging studies, the overall radiologic impression favored a complicated, necrotic inflammatory process — such as thyroid abscess or suppurative thyroiditis — over malignancy. Nonetheless, given the persistent clinical concern raised by the lesion’s size and atypical features, left thyroid lobectomy was planned for both diagnostic clarification and therapeutic management.

The patient deferred surgery for approximately one month for personal reasons. At re-presentation on April 22, 2024, he reported progressive compressive symptoms including worsening dyspnea and dysphagia. Repeat imaging demonstrated marked interval enlargement of the left thyroid mass to 9.2 cm with further extrathyroidal extension and worsening airway deviation (**Figure 2A**). Left thyroid lobectomy was performed on April 22, 2024, under general anesthesia via a standard cervical approach. The operative findings were notable for a firm, heterogeneous, necrotic left thyroid lobe with dense adhesions to the surrounding strap musculature; the plane of dissection was technically challenging owing to extensive perilesional inflammatory change. Grossly negative resection margins were achieved.

**Figure 2:**
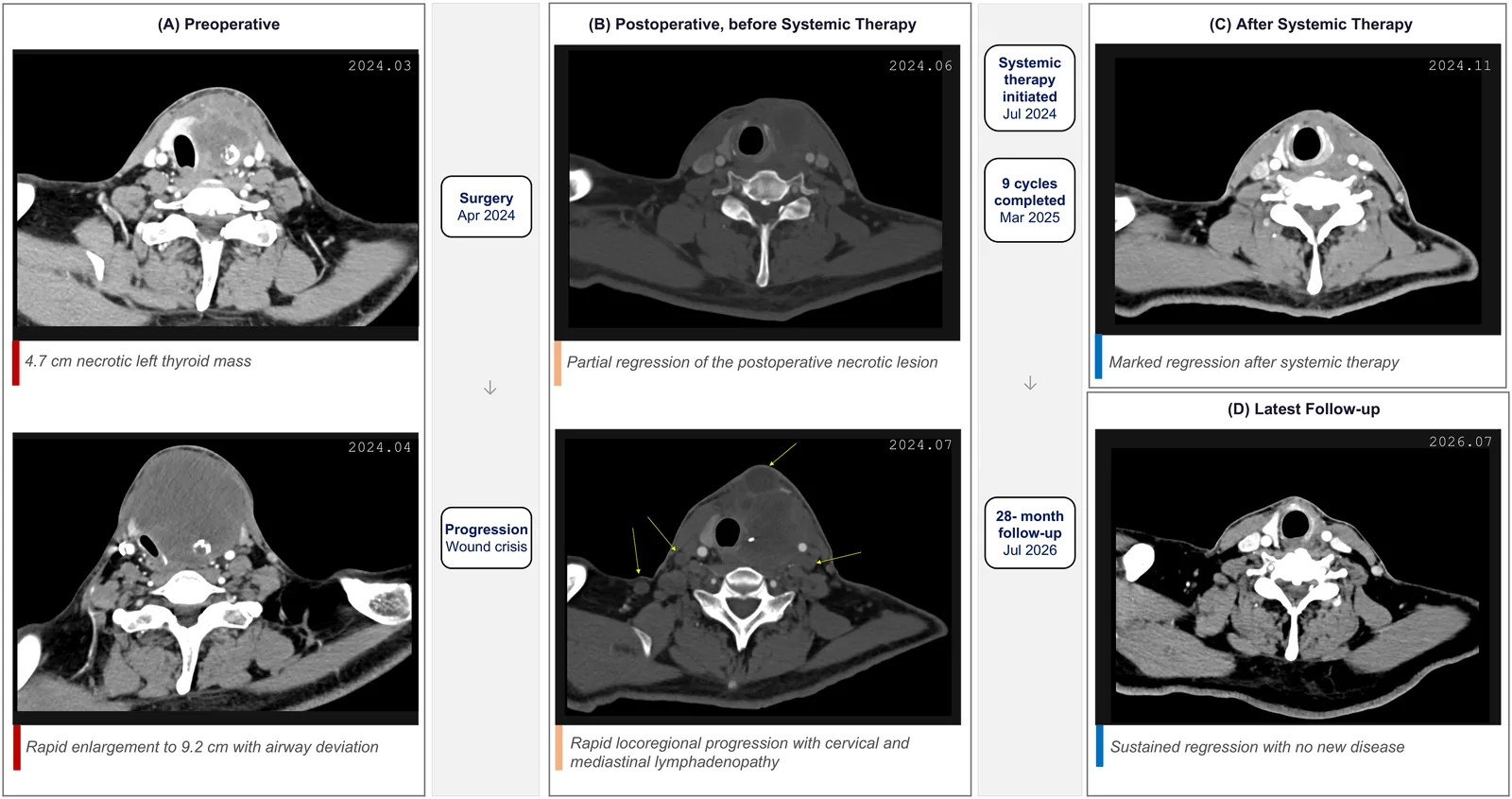
Serial neck CT findings. Representative neck CT images obtained **(A)** preoperatively, **(B)** postoperatively before systemic therapy, **(C)** after systemic therapy, and **(D)** at the latest follow-up (July 2026, 28 months post-surgery), demonstrating sustained regression with no new disease.

### 2.2 Pathologic and molecular findings

The gross specimen weighed 81.0 g (8.5 × 7.2 × 2.6 cm). Histopathology revealed an 8.0-cm ATC spanning the left upper to lower pole, with extensive necrosis, a high mitotic index (25 mitoses/2 mm²) with atypical forms, gross extrathyroidal extension to the strap muscles (pT3b), focal vascular invasion (one focus, confirmed by CD31/CD61), and lymph node metastasis in 1 of 8 perithyroidal nodes (largest focus 0.5 cm, without extranodal extension), yielding pT3bN1a (AJCC 8th edition, Stage IVB at surgery, subsequently upstaged to Stage IVC on postoperative imaging). Resection margins were negative; lymphatic and perineural invasion were absent. Immunohistochemistry demonstrated PAX8 and CK-7 positivity and a Ki-67 proliferative index of 70%; TTF-1, thyroglobulin, and p53 (<10%) were negative.

Given the unexpected diagnosis of ATC, the pathology department immediately notified the surgical team, and an urgent multidisciplinary discussion was convened. To expedite treatment planning, BRAF V600E mutation status was prioritized using PNA-mediated real-time PCR, given its direct therapeutic implications (result: negative). Concurrently, a comprehensive solid tumor NGS panel was ordered to characterize the broader molecular landscape. TERT promoter mutation testing was also negative. NGS identified alterations in CDKN2A (p.Gly111LeufsTer28, 90.0%), TP53 (p.Arg342Ter, 20.3%), NRAS (p.Gln61Arg, 62.2%), ARID1A (p.Gln732Ter, 46.8%), and NBN (p.Ser509IlefsTer2, 64.9%), without actionable mutations, amplifications, or gene fusions. Tumor mutational burden was 4.73 mutations/Mb (microsatellite stable). PD-L1 immunohistochemistry demonstrated a combined positive score (CPS) of 90 by both 22C3 and SP263 clone assays, indicating extreme tumoral and stromal PD-L1 expression. The key clinical and molecular features are summarized in **Table 1**.

**Table 1:** Clinical, pathologic, and molecular features.

Demographics	
Age/Sex	45 years/Male
ECOG performance status	0
Pathology	
Specimen weight	81.0 g (8.5 × 7.2 × 2.6 cm)
Tumor size	8.0 cm
Histology	Anaplastic thyroid carcinoma
pT stage	pT3b (gross ETE to strap muscles)
pN stage	pN1a (1/8 nodes, 0.5 cm, no ENE)
AJCC 8th stage	Stage IVB (surgery) → Stage IVC (post-staging PET/CT)
Resection margins	Negative (R0)
Mitotic index	25/2 mm² (with atypical forms)
Ki-67	70%
Vascular invasion	Present (focal, 1 focus)
Lymphovascular/perineural invasion	Absent
IHC	PAX8+, CK-7+; TTF-1−, Tg−, p53 <10%
Molecular Profiling	
BRAF V600E	Negative (PNA-mediated real-time PCR)
TERT promoter mutation	Negative
NGS significant alterations	CDKN2A (90.0%), TP53 (20.3%), NRAS Q61R (62.2%), ARID1A (46.8%), NBN (64.9%)
Actionable targets	None identified
TMB	4.73 mutations/Mb (MSS)
PD-L1 CPS (22C3 & SP263)	90 — extreme expression
Treatment	
Surgery	Left thyroid lobectomy (April 22, 2024)
Radiotherapy	Not administered
Systemic therapy	Lenvatinib 20 mg/day + Pembrolizumab 200 mg q3w
Cycles administered	9 (July 12, 2024 – March 21, 2025); intervals prolonged before Cycle 4 (+12 days) and Cycle 9 (+~9 weeks)
Reassessment CT (Mar 19, 2025)	Sustained response, no progression; basis for concluding therapy at Cycle 9
Dose reductions/interruptions	None
Outcome (July 13, 2026)	
Neck CT (July 6, 2026)	All negative findings; no new disease
Chest CT	No regrowth of pulmonary metastatic micronodules
Abdominal CT	Near-complete regression of peritoneal seeding
Wound status	Fully healed; mild adhesion-related discomfort at prior drain site, no evidence of recurrence
Survival status	Alive, disease controlled; off systemic therapy

ATC, anaplastic thyroid carcinoma; ETE, extrathyroidal extension; ENE, extranodal extension; AJCC, American Joint Committee on Cancer; IHC, immunohistochemistry; Tg, thyroglobulin; NGS, next-generation sequencing; TMB, tumor mutational burden; MSS, microsatellite stable; CPS, combined positive score; CT, computed tomography.

### 2.3 Postoperative course, staging, and treatment challenges

Postoperative staging PET/CT (May 2024) revealed a probably metastatic left cervical level 4 lymph node and possible peritoneal seeding in the left paracolic gutter. Chest CT (June 2024) demonstrated newly noted nodules in the right lower lobe and left upper lobe suspicious for pulmonary metastases. Abdominal CT (June 2024) identified multiple nodular lesions in bilateral paracolic gutters (largest 1.5 cm) consistent with seeding metastases, formally confirming Stage IVC disease. Thyroid CT (June 2024) showed partial regression of the necrotic lobectomy bed, suggesting a degree of spontaneous local disease control following debulking surgery (**Figure 2B**).

The early postoperative course was complicated by progressive wound deterioration at the operative site, characterized by skin necrosis, persistent serous discharge, and contact bleeding, which precluded early radiotherapy and raised concern for local ATC progression (**Figure 3A**). Surgical re-consultation was obtained; a palpable, protruding soft tissue mass at the operative bed was identified and confirmed to be consistent with rapid tumor regrowth rather than a surgical complication alone. Revision surgery was judged non-beneficial given the biology of ATC and the high likelihood of immediate regrowth. Systemic therapy initiation was further delayed by patient-related factors, including family bereavement during June 2024. During this interval, repeat thyroid CT (July 2024) demonstrated marked interval progression with new bilateral cervical and mediastinal lymphadenopathy and probable left upper lung metastases. The MDT elected not to pursue radiotherapy given the persistent wound and rapidly progressive disease, and determined that immediate initiation of systemic therapy represented the highest clinical priority.

**Figure 3:**
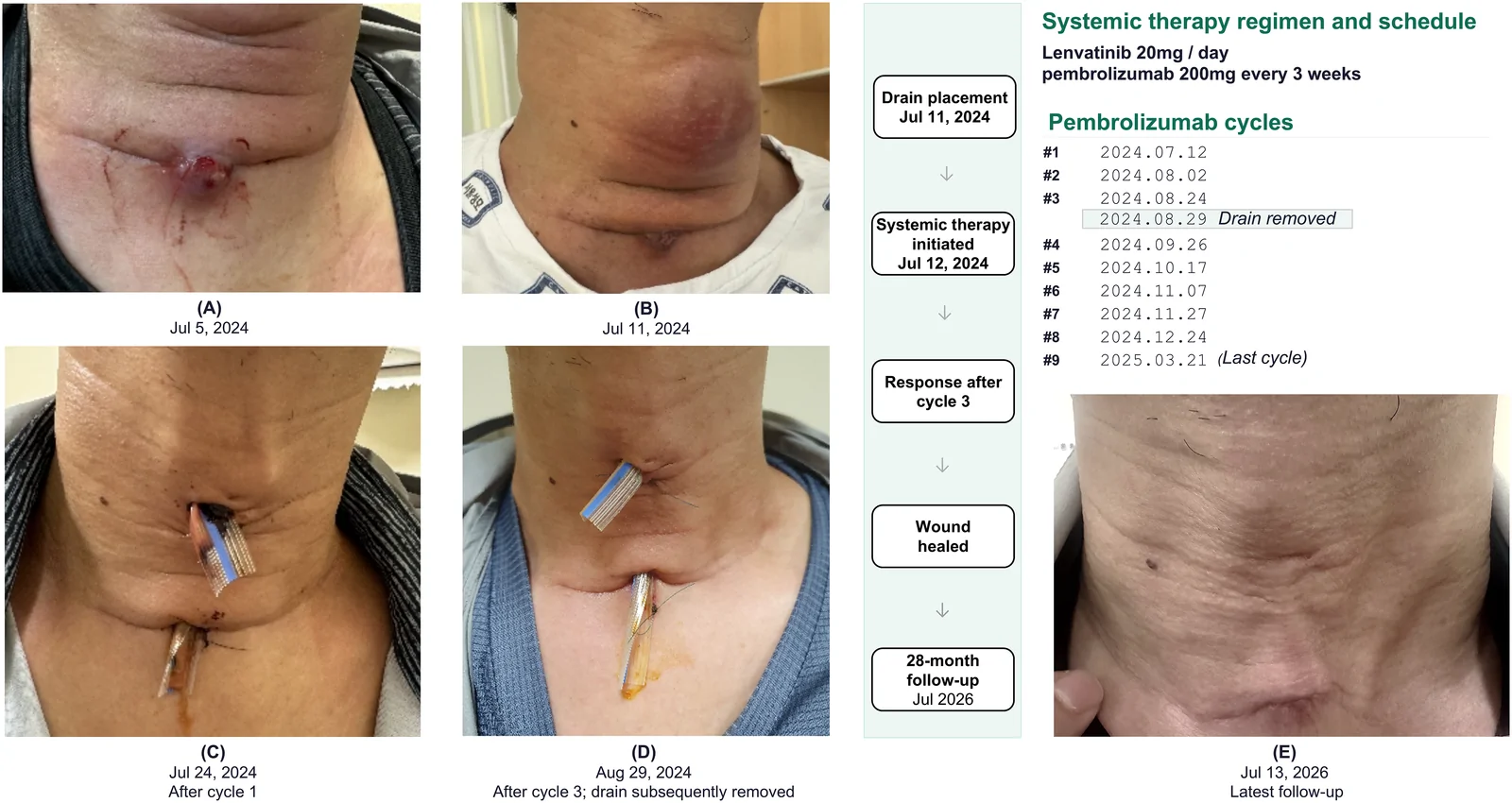
Serial wound photographs with systemic therapy schedule. Sequential wound photographs and systemic therapy schedule, from **(A)** July 5, 2024 and **(B)** July 11, 2024 (drain placement) through **(C)** July 24, 2024 (after cycle 1), **(D)** August 29, 2024 (after cycle 3; drain subsequently removed), and **(E)** July 13, 2026 (latest follow-up, 28 months), demonstrating progressive wound healing alongside the pembrolizumab treatment cycles.

### 2.4 Systemic therapy and concurrent wound management

In the absence of actionable molecular targets and given the extremely high PD-L1 CPS of 90, lenvatinib (20 mg orally once daily) plus pembrolizumab (200 mg intravenously every 3 weeks) was initiated on July 12, 2024, following MDT consensus, based on evidence from the prospective phase II ATLEP trial in BRAF wild-type ATC (2). Nine cycles were administered from July 12, 2024 to March 21, 2025. While most cycles were delivered at the standard 3-week interval, two cycles were delayed relative to schedule (Cycle 4, by approximately 12 days; Cycle 9, by approximately 9 weeks), related to the patient’s difficulty maintaining the treatment schedule because of financial and personal circumstances (see Patient Perspective).Shortly after lenvatinib initiation, the operative wound transiently worsened, with increased swelling, serosanguineous discharge, and contact bleeding, consistent with the known anti-angiogenic effect of lenvatinib on wound healing. Drain insertion and surgical decompression were performed on July 11, 2024 (**Figure 3B**). The fundamental MDT decision was to maintain systemic therapy without interruption or dose reduction while the surgical team managed the wound in parallel. This required close coordination between the thyroid surgery and medical oncology teams, with daily or alternate-day wound dressing changes maintained both during inpatient admission and in the outpatient setting post-discharge. No antifungal prophylaxis was administered; no infectious wound complications were identified.

Following three cycles, the patient demonstrated a favorable clinical and radiologic response. By August 29, 2024, wound discharge had resolved, the drain was removed, and the wound was confirmed to be in a healing state on surgical outpatient review (**Figure 3D**). Regular wound surveillance was discontinued, and complete wound epithelialization was achieved during ongoing systemic therapy. No dose reductions of lenvatinib or pembrolizumab were required throughout the entire treatment course. Repeat cross-sectional imaging of the neck, chest, and abdomen/pelvis on March 19, 2025, two days before the final cycle, confirmed a sustained treatment response with no evidence of disease progression. In view of this response, together with the patient’s ongoing difficulty continuing therapy for financial and personal reasons (see Patient Perspective), the multidisciplinary team and patient jointly elected to conclude systemic therapy after Cycle 9 (March 21, 2025) rather than continue treatment indefinitely.

### 2.5 Clinical outcome and follow-up

At the most recent follow-up on July 13, 2026 — 28 months after initial presentation and 16 months following completion of the last cycle of systemic therapy — comprehensive imaging demonstrated durable disease control across all previously affected sites. Neck CT obtained one week earlier (July 6, 2026) showed no newly developed abnormality, with small, stable residual lymph nodes in the left level 4 and paratracheal regions, unchanged from prior studies (**Figure 2D**). Chest CT demonstrated no regrowth of previously regressed metastatic micronodules in the left upper lobe and right lower lobe. Abdominal CT showed near-complete regression of the peritoneal seeding metastasis, with no newly developed seeding lesions identified. On outpatient examination the same week, the patient remained clinically well with a fully healed neck wound (**Figure 3E**); he reported mild residual discomfort related to skin adhesion at the site of prior drain placement — a localized sequela of wound healing rather than a sign of disease activity or recurrence (**Figure 2**). The patient remained alive with maintained performance status and no evidence of clinical disease progression. No further systemic therapy has been administered since Cycle 9 (March 21, 2025). The complete clinical timeline is summarized in **Figure 1**.

## 3 Discussion

This case report illustrates that prolonged disease control is achievable in selected patients with metastatic BRAF wild-type ATC through individualized multimodal management, and highlights three interrelated themes: the prognostic relevance of the molecular profile, the potential role of extreme PD-L1 expression as a biomarker for immune checkpoint inhibitor selection, and the indispensable contribution of MDT-coordinated surgical wound management to treatment continuity and outcome.

Survival beyond 2 years in metastatic ATC represents a clinically meaningful threshold that was historically achieved only rarely. In the largest reported ATC cohort — 360 patients from Memorial Sloan Kettering Cancer Center (MSK) — the 2-year and 5-year OS rates were 17% and 11%, respectively (9, 10). A subsequent MSK case-control study comparing 46 long-term survivors (LTS, ≥2 years) with 75 controls identified surgical resectability, low pretreatment neutrophil-to-lymphocyte ratio, absence of BRAF/RAS–TERT promoter co-mutation, and low tumor-infiltrating myeloid-derived suppressor cells as independent predictors of LTS (11). Our patient fulfilled several of these criteria: primary tumor resection was achieved with negative margins, the molecular profile was BRAF wild-type and TERT-negative, and the patient was relatively young (45 years) with an intact performance status. These features collectively defined a favorable prognostic profile that likely contributed to the observed outcome.

The absence of BRAF V600E mutation, confirmed by PNA-mediated PCR, while precluding dabrafenib plus trametinib — which achieves an ORR of 56% in BRAF-positive ATC (4, 5) — defined a molecular subtype with distinct biological behavior. The co-occurring NRAS Q61R alteration (62.2% variant allele fraction) with disruptions in TP53 and CDKN2A is consistent with the genomic landscape of BRAF wild-type ATC, yet the absence of TERT promoter co-mutation is particularly notable. Large-scale genomic analyses have demonstrated that concurrent RAS and TERT mutations confer synergistically worse prognosis, implying that single-gene or wild-type TERT status may be associated with relatively more indolent tumor biology in the context of ATC (9, 10).

The PD-L1 CPS of 90 was the principal immunological rationale for pembrolizumab selection. PD-L1 expression has been identified in 22–29% of ATC tumors at conventional positivity thresholds, but extreme overexpression as observed in this patient is unusual and clinically significant. Case reports have documented complete metabolic responses to pembrolizumab monotherapy in BRAF-negative ATC patients with PD-L1 TPS approaching 90–100% (6, 7). In the prospective phase II ATLEP trial — to date the largest prospective study of lenvatinib plus pembrolizumab in BRAF wild-type ATC — the clinical benefit rate was 96.3%, median OS was 10.25 months, and 25.9% of ATC patients survived beyond 2 years (2). Crucially, all patients achieving durable or complete responses demonstrated PD-L1 TPS >50% or tumor mutational burden >5 mutations/Mb (2). Our patient’s PD-L1 expression was assessed using the combined positive score (CPS), which incorporates both tumoral and immune cell staining and is not directly interchangeable with TPS; TPS was not separately assessed in this case, and direct comparison with the TPS-based threshold reported in ATLEP should therefore be interpreted with caution. Nonetheless, a CPS of 90 reflects an extremely high level of PD-L1 expression by any scoring convention, and may offer a plausible, though currently exploratory rather than validated, biomarker rationale for the sustained response observed; harmonized PD-L1 scoring across future studies will be needed to clarify its predictive value in ATC. A subsequent real-world single-center experience in 36 patients with BRAF wild-type ATC treated with lenvatinib plus pembrolizumab outside a clinical trial confirmed meaningful clinical activity in routine practice, with a confirmed objective response rate of 36% (including complete response in 9% of patients) and a median duration of response of 16.0 months, although median overall survival in this more heavily pretreated real-world cohort was shorter (7.7 months) than in the original trial population (12).

A critical and often underappreciated determinant of the outcome in this case was the active surgical management of postoperative wound complications during ongoing systemic therapy. Lenvatinib is a multi-targeted tyrosine kinase inhibitor with potent anti-angiogenic activity across VEGFR1–3, FGFR1–4, PDGFRα, RET, and KIT; its inhibition of VEGFR-mediated angiogenesis impairs wound healing and increases the risk of hemorrhagic and fistula-related complications. In the ATLEP trial, grade 3–4 hemorrhagic or fistula events occurred in 11% of ATC patients (2). In our case, wound deterioration following lenvatinib initiation was managed through drain insertion, iterative surgical wound care, and sustained close follow-up by the surgical team — all conducted without interrupting or reducing systemic therapy. This strategy, consistent with ATA 2021 guidelines prioritizing treatment continuity through coordinated MDT care (1), may have been the critical intervention enabling the continued antitumor effect that led to wound healing and subsequent disease control. From a surgical perspective, this case underscores that the role of surgery in ATC extends beyond the initial resection and encompasses active postoperative wound surveillance and intervention throughout the systemic therapy course.

This report carries the inherent limitations of a single-patient observation. Causal attribution of survival benefit to any individual therapeutic component is not possible, selection bias cannot be excluded, and residual stable nodal disease on follow-up imaging precludes conclusions regarding cure. Nevertheless, this case adds to the growing body of evidence that a nihilistic approach to ATC is no longer universally warranted. Carefully selected patients — particularly those presenting with extreme PD-L1 expression, BRAF/TERT wild-type molecular profiles, preserved performance status, and resectable primary disease — may derive meaningful and durable benefit from individualized multimodal therapy incorporating immune checkpoint inhibition. Systematic prospective collection of molecular and clinical data from ATC patients achieving long-term survival is urgently needed to validate these predictive biomarkers and refine patient selection.

Additionally, formal RECIST 1.1-based tumor response assessment was not performed prospectively in this case; treatment response was assessed qualitatively by the treating radiologists and multidisciplinary team based on serial cross-sectional imaging, precluding calculation of best overall response, time to response, or duration of response according to standardized criteria.

## 4 Conclusion

Durable disease control beyond 2 years is achievable in selected patients with metastatic BRAF wild-type ATC. In this case, extreme PD-L1 expression (CPS 90) informed the selection of lenvatinib plus pembrolizumab, active multidisciplinary surgical wound management enabled uninterrupted treatment continuity despite significant wound complications, and a favorable molecular profile (BRAF/TERT wild-type) supported the biological plausibility of the sustained response. These findings reinforce the imperative of comprehensive molecular profiling at diagnosis, early MDT engagement, and individualized multimodal management in all patients with ATC.

## 5 Patient perspective

Partway through the treatment course, the patient considered discontinuing systemic therapy because of the financial burden of prolonged treatment and difficulty accommodating the therapy schedule around personal and work-related obligations, which contributed to delays before some treatment cycles. Active encouragement and support from the multidisciplinary care team, including the thyroid surgery and medical oncology teams, addressed these concerns and reinforced the potential benefit of continuing treatment. This support was instrumental in his decision to persevere, and he ultimately completed nine cycles of systemic therapy. Reassessment imaging shortly before the final cycle confirmed a sustained treatment response, and in view of this response together with the patient’s ongoing difficulty continuing therapy, the patient and multidisciplinary team jointly decided to conclude systemic therapy after Cycle 9 rather than continue indefinitely. In retrospect, the patient has expressed that this decision allowed him to achieve durable disease control and return to daily activities.

## Data Availability

The original contributions presented in the study are included in the article/supplementary material. Further inquiries can be directed to the corresponding author.
